# Is home-based self-swabbing feasible for postoperative wound culture after cardiac surgery? A multicentre mixed-methods feasibility study in the UK

**DOI:** 10.1136/bmjopen-2025-112691

**Published:** 2026-02-10

**Authors:** Melissa Rochon, Judith Tanner, Karen Cariaga, Roy Harris, Keith Wilson, Chris Newby, Kumbi Kariwo, Luciana Sowole, Sarah J Bolton, Janet Bouttell, Ishtiaq Ahmed

**Affiliations:** 1 Directorate of Infection, Guy's and St Thomas’ NHS Foundation Trust, London, UK; 2 School of Health Sciences, University of Nottingham, Nottingham, UK; 3 Research Support Services (Leicester Hub), University of Nottingham, Nottingham, UK; 4 Research Department (ICECAP), Liverpool Heart and Chest Hospital NHS Foundation Trust, Liverpool, UK; 5 Equality Inclusion and Diversity, Birmingham Community Healthcare NHS Foundation Trust, Aston, UK; 6 Directorate of Infection, Guy's and St Thomas’ Hospitals NHS Trust, London, UK; 7 CHEATA, Nottingham University Hospitals NHS Trust, Nottingham, UK; 8 Department Cardiac Surgery, University Hospitals Sussex NHS Foundation Trust, Worthing, UK

**Keywords:** Feasibility Studies, SURGERY, MICROBIOLOGY, Self-Management

## Abstract

**Introduction:**

Poor access to surgical wound swabbing in the community often results in delayed or inappropriate antibiotic prescribing for surgical site infections. This delay can contribute to prolonged wound healing and poor antimicrobial stewardship. Patient self-swabbing at home could improve access to diagnostic testing, but its feasibility and acceptability remain unexplored.

**Methods and analysis:**

TREASURE is a multicentre, mixed-methods feasibility study. A total of 40 patient participants and 10 staff stakeholders will be included. 40 adult patients undergoing cardiac surgery via median sternotomy will be recruited from Harefield Hospital (n=25) and the Royal Sussex County Hospital (n=15). Eligible participants will receive a coproduced self-swabbing set of instructions and kit at discharge and perform wound swabbing at home within 1–21 days, observed remotely by a researcher via Microsoft Teams. Swabs will be couriered to a central laboratory for bacterial culture with antimicrobial susceptibility testing for pathogens.

The primary feasibility outcome is the proportion of patients successfully completing self-swabbing at home to obtain usable culture swabs with samples received at the laboratory within 24 hours and deemed suitable for processing. Secondary safety and acceptability outcomes include usability of the kit and instructions; patient satisfaction; viability of samples for laboratory analysis; and recruitment and retention rates. A 30-day follow-up will capture wound complications, antibiotic prescribing and healthcare utilisation via patient questionnaires, case note review, general practitioner confirmation and patient interviews. 10 staff stakeholders will be interviewed to inform pathway development.

Quantitative data will be analysed descriptively, with proportions reported alongside 95% CIs. Qualitative data from patients will undergo thematic analysis, and stakeholder interviews will be coded using Normalisation Process Theory. An early health economic model will be developed to explore resource use, costs and proportions of appropriate and timely antibiotic use between current pathways and a proposed pathway, including self-swabbing.

**Ethics and dissemination:**

West of Scotland Research Ethics Service has reviewed and approved the study (REC reference: 25/WS/0079). Findings will be disseminated through the study website, a webinar, peer-reviewed publications, conference presentations, patient and public involvement-led activities and engagement with National Health Service (NHS) stakeholders.

**Trial registration numbers:**

NCT07200401, ISRCTN28466609.

**STRENGTHS AND LIMITATIONS OF THIS STUDY**

- The study uses a mixed-methods feasibility design incorporating quantitative process measures and qualitative interviews to assess the practicality of the intervention.
- Patient and public involvement was integrated into the development of study materials, including the swab kit design and instructions, to ensure methodological appropriateness.
- Multiple data outcomes (recruitment, adherence, acceptability and laboratory processing metrics) will be collected to evaluate key feasibility parameters needed for a future trial.
- Conducting the study within a single surgical specialty and across two hospital sites may limit generalisability.

## Introduction

 Surgical wound complications remain a significant clinical and public health challenge, affecting patient recovery, healthcare costs and antimicrobial resistance (AMR). Poor access to wound swabbing in the community frequently results in patients receiving delayed or inappropriate antibiotic treatment for surgical site infections (SSIs).^1 2^ Such delays can exacerbate complications, prolong healing and contribute to the global threat of AMR. While patient self-swabbing at home offers a practical means of improving diagnostic access, its feasibility and effectiveness have not yet been explored.

Most SSIs develop after patients have been discharged from hospital, with the majority being classed as superficial infections.^3 4^ In the UK, current standard practice at discharge is to advise patients to contact their general practitioner (GP) if wound concerns arise. Increasingly, patients are also monitored remotely via telephone calls, text messages, emails or virtual consultations.^5^ However, when infection is suspected, patients may be required to attend either their GP surgery or hospital for wound review and, if deemed appropriate, specimen collection (swabbing).

Swab cultures can aid the identification of significant organisms and guide the choice of antibiotic, supporting a move away from empiric broad-spectrum prescribing towards narrower, targeted therapy.^6^ This serves to increase the likelihood of effective treatment and reduce unnecessary broad-spectrum antibiotic exposure, which in turn mitigates the development of AMR. While wound swabbing is not required in every case and clinical assessment remains central, timely access to culture results provides clinicians with valuable information to optimise care and stewardship.^7^

Several barriers restrict timely access to wound swabbing. These include: difficulty obtaining GP appointments^8^ and the inconvenience of hospital visits, which may involve additional costs, travel, carer support and time away from work. These challenges raise important questions: how can wound infections be identified earlier; could point-of-care testing in primary care improve outcomes and reduce unnecessary antibiotic use; and what role could patient self-care and carer involvement play in infection management?^9^

The scale of the problem is considerable. Data from 21 patients across six cardiothoracic centres indicate that only 2% of patients reporting wound problems following cardiac surgery underwent swabbing, while only 28% of those prescribed antibiotics for wound complications had a swab taken.^10^ Of the approximately five million surgical procedures performed annually in England, an estimated one million patients (20%) develop a wound complication, with around 250 000 (5%) experiencing a wound infection.^11^ These complications can require additional surgery, may take months or years to resolve, negatively impact quality of life^12^ and contribute to an estimated annual cost to the NHS of £1 billion, with individual cases costing up to £100 000 to manage.^13 14^ The UK Health Security Agency (UKHSA) reported in 2018 that 20% of antibiotics prescribed in primary care were inappropriate.^15^ Inappropriate antibiotic use further compounds the risk of AMR, delays recovery and increases mortality and healthcare expenditure worldwide.^16^

International and national policy and research priorities highlight the importance of addressing this issue. In 2023, the WHO launched a global AMR forum to tackle this threat to planetary health.^17^ In the UK, the Royal College of General Practitioners called for better access to point-of-care testing to support accurate diagnosis and treatment of infections and the James Lind Alliance, which brings together patients, carers and clinicians, identified infection diagnosis and management as a key research priority.^9^ Furthermore, the NHS Long Term Plan emphasises improving service accessibility and supporting safe, convenient care at home,^18^ while commitments to reduce patient travel align with the NHS target of achieving carbon net zero by 2040.^19^

There is an established precedent for self-swabbing. Self-swabbing and testing for Covid were quickly and successfully implemented nationally in the UK.^20^

Against this backdrop, the potential for home-based surgical wound swabbing warrants urgent investigation as a strategy to improve timely diagnosis, optimise antibiotic prescribing, reduce AMR and enhance patient experience.

## Methods and analysis

### Study design

This is a multicentre, mixed-methods non-randomised feasibility study with the aim of evaluating the safety, acceptability and potential cost-effectiveness of patients self-swabbing surgical wounds at home. At hospital discharge, 40 adult patients across two hospital sites having had cardiac surgery will be given a self-swabbing kit and a set of instructions, coproduced by a patient and public involvement (PPI) group. All participants continue to receive usual postoperative follow-up and access to standard services as per site practice. Patients will be observed swabbing their surgical wounds at home by a researcher via Microsoft (MS) Teams and follow-up interviews will be conducted. Quantitative data will assess feasibility, safety, usability and clinical outcomes, while qualitative interviews with patients and staff stakeholders will explore acceptability and implementation.

An early health economic model will be developed alongside the clinical study to inform the wider research programme about the potential costs and impacts on antibiotic prescribing of introducing self-swabbing into the clinical pathway following cardiac surgery.

**Figure 1:**
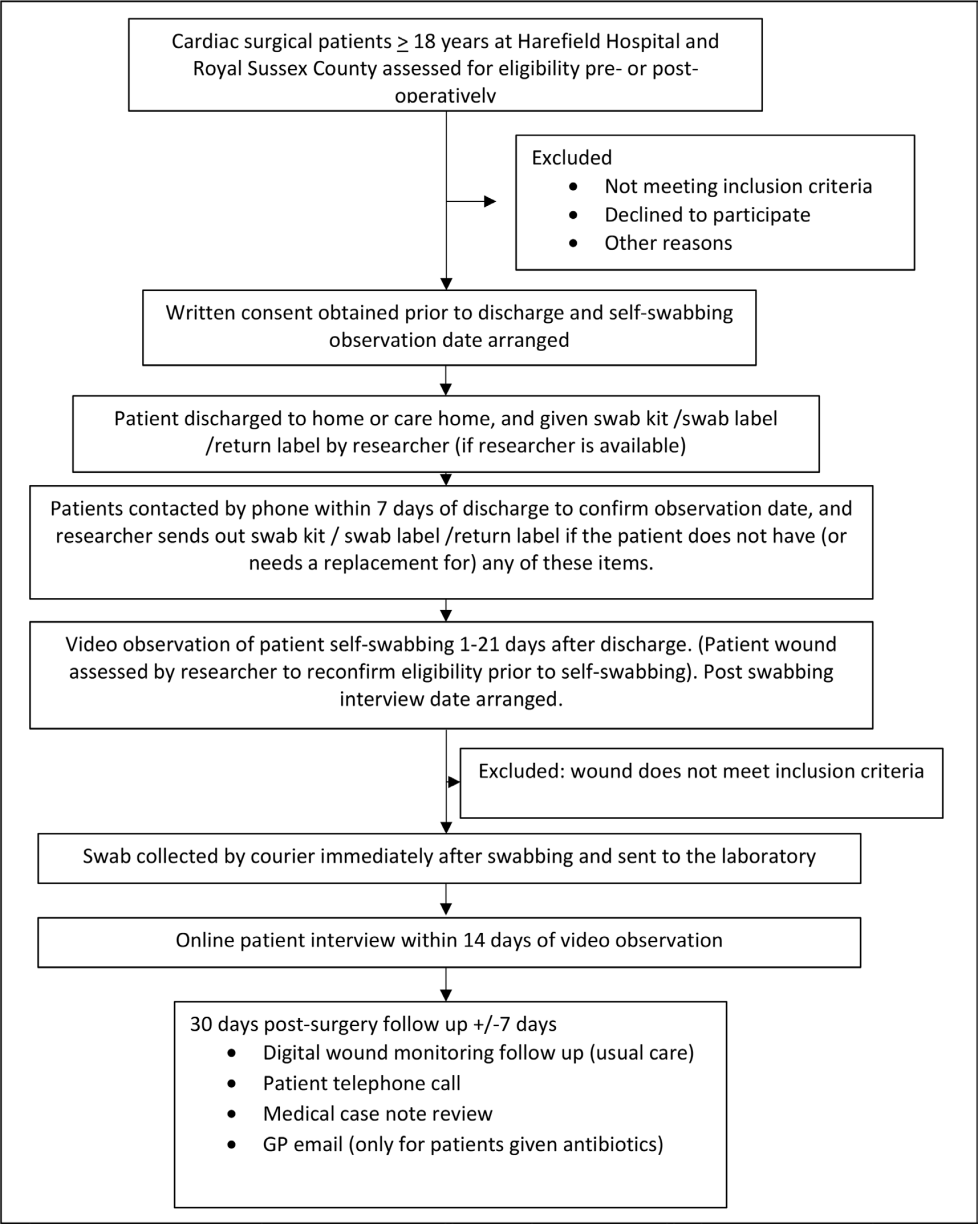
Study flow diagram. GP, general practitioner.

The study will run from January 2026 to May 2026, with participating sites open for recruitment from January to February. The protocol is reported using the Standard Protocol Items Recommendations for Interventional Trials 2025 checklist.^21^ The study flow chart is shown in figure 1.

### Study setting

The two cardiac centres chosen as research sites provide diversity in patient ethnicities, socioeconomic status and geographical location. Harefield Hospital (London Borough of Hillingdon) and the Royal Sussex County Hospital (coastal location) each perform approximately 1000 cardiac operations each year.^22^

### Study participants

Cardiac surgery was selected as the sample population as wound complications and infections are relatively common within this specialty and are among the most expensive to treat.^3^ A total of 40 adult patients (≥18 years) undergoing cardiac surgery via median sternotomy will be recruited: 25 from Harefield Hospital, Uxbridge and 15 from the Royal Sussex County Hospital, Brighton, UK. Additionally, patients who would like to take part in the study but require assistance can join along with an adult carer with the consent of both patient and carer. See onlinesupplemental materials 1  2 for participant and carer consent forms. Equipment (connected device and data allowance) will be provided for patients without internet access.

### Participant inclusion and exclusion criteria

Patients will be eligible to take part if they meet the following criteria:

- Cardiac surgery ≥18 years old patients with a central chest wound, where the wound is a closed wound—assessed by the researcher.
- Patients having elective or urgent surgery.
- Patients who have been discharged home or to a care home.
- Willing and able to provide written informed consent prior to participation in the clinical investigation.
- Willing and able to comply with all study-related procedures, with digital resource/internet access.

Patients will be excluded for the following reasons:

- Cardiac surgery patients with open wounds extending beyond skin level, or where deep tissue, organs or implants are visible, wounds with constant or heavy discharge of fluid, wounds leaking pus at the time of the video consultation.
- Patients with a dressing covering their wound at the time of the video consultation.
- Congenital or acquired immunodeficiency, bone marrow disease, diabetes, autoimmune conditions requiring immunosuppressive treatment, any immunosuppressive medication at the time of consent or within the last 4 weeks before consent due to potential issues with wound healing.
- Undergoing active cancer treatment at the time of consent or planning to start cancer treatment within the study period or completed cancer treatment within the last 4 weeks of the study commencing due to potential issues with wound healing.

### Sample size

As a feasibility study, no formal hypothesis testing is planned. Recruitment of 40 patients will allow estimation of a 90% successful self-swabbing rate with a 95% CI of 76% to 97%, calculated using the exact binomial method. A 90% success rate for self-swabbing was selected as the predefined ‘green-light’ criterion to justify progression to a full trial.

### Recruitment and consent

For planned cardiac surgery, potential participants on the elective surgical listing will be identified by the direct care team and will be told about the study at outpatients’ appointments.

For urgent cardiac surgery or transferred patients, potential participants on the urgent/transferred patient surgical listing will be identified by the direct care team and will be told about the study on the ward.

Potential participants will be identified by the direct care team and given a flyer containing brief information about the study. Verbal consent to share contact details with a member of the research team will be sought from patients who express an interest in taking part, which will be documented in the medical notes and on the screening log. Details of interested participants will be relayed to the researcher via email. Patients who express an interest in taking part will be telephoned by the researcher to discuss the study, answer any questions and will be sent a participant information sheet (PIS) via post or email, based on their preference. Individuals will be given a minimum of 24 hours to consider participation in the study.

Consent for collecting and use of wound culture samples will be obtained using the study informed consent form (ICF). Samples will not be stored for future research past the end of this study. This is stated in the ICF and PIS.

Each participant will be reimbursed £40 for their time plus £5 to cover internet costs. Participants can choose between cash paid into their bank account or an Amazon voucher.

### Withdrawal criteria

Participation in this study is voluntary. Patients are not required to participate and may decide to withdraw at any time. Participants may be discontinued from study participation on request, through non-compliance, through loss to follow-up, or if they are deemed ineligible after consent has been obtained.

Participants who no longer wish to participate can contact the researcher using the contact details provided in the PIS. Any data submitted prior to withdrawal will still be used in the study. Patients will no longer receive any contact from the research study, although they will continue to receive standard surgical care follow-up.

Study data already recorded in patient records for non-responding participants will be kept unless they explicitly withdraw their consent. Discontinued participants will be assessed for adverse events (AEs) at the final study-required contact (days 28–30) with continuing assessment of procedure-related AEs until these are resolved or stable (ie, unlikely to resolve or managed successfully with treatment). Attempts will be made to replace participants who withdraw post consent, are lost to follow-up or do not meet final eligibility criteria.

### Stakeholder interviews

Attempts will be made to replace staff who withdraw from the stakeholder interview.

### Intervention

At discharge, eligible patients will be provided with a swab kit containing two culture swabs, two ampoules of saline, three alcohol-free hand wipes, a packet of gauze, a return envelope and rigid packaging, two labels, a pen and a set of instructions. At a prearranged appointment time convenient to the participant and researcher, and within 1–21 days postdischarge, patients will perform self-swabbing assessed remotely by a researcher (via Microsoft (MS) Teams). The researcher will undertake all MS Teams meetings in a private setting with a locked door to maintain participant privacy and confidentiality. After checking, the wound edges remain closed and non-infected so that the patient still meets the study eligibility criteria. The researcher will use a checklist to record whether the participant is able to comply unaided with each step of the instructions, while maintaining the sterility of the swab. The researcher will also ensure infection prevention procedures are followed and provide guidance if needed. A distress protocol will be available, as required.

The researcher will arrange for completed swabs to be collected as soon as possible after swabbing and couriered to an independent UK laboratory (based in Basildon), used by the NHS. In the 2 weeks following the observed swabbing, participants will take part in a semistructured online interview exploring acceptability and usability of self-swabbing, the kit and the instructions. Interviews, conducted by the researcher or delegated member of the research team, will be audio-recorded, transcribed verbatim, pseudo-anonymised and analysed using thematic analysis.^23^

### Follow-up and data collection

Follow-up data will be collected at 30 days postoperation. Patients’ wounds will be assessed for infection or complication using the UKHSA wound monitoring questionnaire (and image review) via digital monitoring. The researcher will telephone the patient to collect any readmission, treatment or use of NHS resources data. The GPs of patients prescribed antibiotics will be contacted to obtain prescribing details. Medical case note review will be undertaken to identify those with confirmed evidence of wound infection and the management strategy and antibiotics used. The time taken to transport swabs to the laboratory will be documented as well as the condition of the wound swabs on arrival (usability). Samples will be processed for culture and antimicrobial susceptibility testing to confirm their viability. The results of these study swabs will not be shared with participants or the surgical team routinely. Data will be stored using Research Electronic Data Capture (REDCap), with full audit trails. AEs reviews will take place at any time point throughout the study duration.

#### Staff stakeholder interviews

Ten stakeholders will be interviewed. Stakeholders are people who contribute to or influence the usual care surgical wound follow-up pathway and will be identified through stakeholder mapping. Stakeholders from the two participating sites will likely include surveillance staff, infection and wound care nurses, microbiologists and surgeons. In addition, other stakeholders, including GPs, laboratory staff and representatives from companies that make self-testing kits and commissioners will also be approached. The usual care team and the research team will use convenience sampling to invite individuals for interview that represent stakeholder professional groups. The focus of the interviews is to explore how self-swabbing at home can fit into the existing pathway. Stakeholder interviews will be conducted online by the researcher, audio-recorded, transcribed verbatim, pseudo-anonymised and analysed using Normalisation Process Theory.^24^

## Outcomes

### Primary outcomes

Feasibility of home-based wound self-swabbing, defined as the proportion of patients completing the procedure successfully (ie, compliance with swabbing instructions) with samples received at the laboratory within 24 hours, and deemed suitable for processing.

Safety of the process, assessed through adherence to swabbing protocol, AEs and participant-reported concerns.

Acceptability of the swabbing process, assessed through Likert scale ratings and qualitative interviews.

### Secondary outcomes

- Acceptability of the swabbing instructions and kit, assessed through Likert scale ratings and qualitative interviews.
- Usability of swabs, including courier transport times and number of viable swabs for testing.
- New swabbing at home pathway, generated from stakeholder interviews and cost-effectiveness work.
- Recruitment and retention rates, adherence and completeness of data, including any barriers to participation and demographics.

### Data analysis

Quantitative data will be analysed descriptively. Continuous normally distributed variables will be summarised as means and SD; non-normal variables as medians and IQRs; categorical data as counts and percentages. Missing data will be presented as counts and percentages.

A Consolidated Standards of Reporting Trials (CONSORT) diagram will be created to describe patient flow.

The proportion of successful self-swabbing with 95% CI in our feasibility study will inform a sample size calculation for a full clinical trial, along with data from audits and the literature. Qualitative patient interviews will undergo thematic analysis, while stakeholder interviews will be coded using Normalisation Process Theory to assess potential integration into the NHS usual care pathway.

### Pathway development

Pathway development within the TREASURE study will be informed by stakeholder interviews and early cost-effectiveness analyses. Insights from clinicians, managers and patients will be used to map existing care pathways, identify inefficiencies and define where the intervention could add value. The cost of the intervention will be estimated and incorporated into an early health economic model to support assessment of feasibility, value for money and scalability within routine care.

### Health economic modelling

An early health economic model will be developed alongside the clinical study to explore the impact of introducing a self-swabbing aspect into the clinical pathway of follow-on care postsurgery. A cost consequence analysis will compare costs and outcomes over a short time horizon from an NHS health and personal social services perspective. Outcomes of interest will be resource use and costs, including cost of intervention collected during the clinical study and proportions of patients receiving appropriate antibiotics on a timely basis. The standard of care pathway will be set out using data from the literature with any gaps populated using expert opinion. A deterministic approach will be taken, and sensitivity and threshold analysis will explore the impact of individual parameters on costs and outcomes.

## Data protection and management

Data will be handled in accordance with the Data Protection Act 2018, the UK Policy Framework for Health and Social Care Research, and Research Ethics Committee requirements. Participants will be assigned a unique study identifier and identifiable information will be stored separately and securely from study data.

Study data will be collected using REDCap, a secure, password-protected database hosted at Guy’s and St Thomas’ NHS Foundation Trust. Case report form/electronic CRF (CRFs/eCRFs) will not bear the subject’s name or other personal identifiable data. The subject’s study Identification Number (ID) will be used for identification. Access will be restricted to authorised study staff, with audit trails and validation checks to ensure data quality. Once data entry and cleaning are complete, the database will be locked prior to analysis. Pseudo-anonymised datasets will be securely transferred to the study statistician for analysis.

Qualitative interviews will be audio-recorded with consent and transcribed by an approved transcription service. Transcripts will be pseudo-anonymised, analysed and stored securely on the University of Nottingham servers.

Paper records containing identifiable information (eg, consent forms) will be kept in locked cabinets within restricted-access areas at participating hospitals. Electronic and paper records will be archived in line with sponsor procedures; essential documents will be retained for at least 5 years, and electronic interview data will be destroyed after 10 years.

The end of trial will be defined as the database lock, once all participants have completed all the study-related visits and the data has been entered in the eCRF and queries resolved. Data quality and overall study conduct will be undertaken throughout the study by the Trial Steering Committee (TSC) in conjunction with the Sponsor.

To minimise the risk of patients introducing infection into their wounds, only patients with closed and non-infected appearing wounds will be included. The entire process of self-swabbing, including hand hygiene steps, will be supervised by the researcher. Given the relatively low-risk nature of this feasibility study, Data Monitoring Committee responsibilities will be undertaken within the TSC. Safety reporting will be performed as specified in the protocol. The TSC will safeguard the interests of participants and ensure that the trial is conducted to the highest standards of research governance.

## Patient and public involvement

The design of this study is informed by two surveys conducted at Guys and St Thomas’s NHS Foundation Trust with 100 and 46 patient respondents, respectively, focusing on patient priorities, acceptability, practicality and privacy issues.^25^

In the first survey, 75% of respondents said they would be supportive of swabbing their own wounds at home, showing support for this proposal. In the second survey, Patients also said they would welcome the convenience of swabbing at home, but there were concerns that they might disturb the wound healing process, contaminate the wound or not carry out the swabbing correctly. They identified a need for clear instructions.

In response to this, a co-designed set of instructions was developed. A group comprising seven patients with diverse demographics, two consultant microbiologists, a consultant tissue viability nurse, the co-chief investigators and the project manager collaborated over the course of five meetings. Together, they created the swabbing kit contents and instructions for surgical wound swabbing using gel swabs (see onlinesupplemental materials 1  2).

The study team includes a highly experienced PPI representative and a specialist in inclusion, who have both contributed to the design of this study. The PPI representative will chair a PPI group that will contribute to the design and management of the study, including patient-facing documents, developing the dissemination strategy, contributing, delivering and co-producing material for the website, social media, webinar, conference presentations and papers, commenting on study progress and data analysis, and contributing to the final report. An Equality Impact Assessment will help maximise engagement opportunities among disadvantaged groups.

## Ethics and dissemination

West of Scotland Research Ethics Service has reviewed and approved the study (approval date: 04/08/2025; .REC reference: 25/WS/0079) and will be conducted in compliance with the Declaration of Helsinki. Any amendments to the TREASURE protocol will be submitted for review and approval by the Research Ethics Committee (REC), the Sponsor and relevant NHS R&D departments before implementation. This study is sponsored by Guy’s and St Thomas’ NHS Foundation Trust with support from the Royal Brompton & Harefield Hospitals Research Office. For any amendments to the study, the Chief Investigator or designee, in agreement with the Sponsor, will submit information to the appropriate body in order for them to issue approval for the amendment. The Chief Investigator or designee will work with sites (R&D departments as well as the study delivery team) to confirm ongoing Capacity and Capability for the study.

In line with good clinical practice guidelines for observational studies, written consent will be obtained by appropriately trained researchers or clinicians. Participants are free to decline involvement without giving a reason, although where reasons are given, these will be collected as part of screening data. If participants do not wish to participate, they will continue to receive standard postoperative care, and their standard of care will not change.

The chief investigators and coapplicants have no financial interests in the intervention.

Study findings will be reported using the CONSORT checklist as the main framework, supplemented with relevant CONSORT-Pilot items to highlight feasibility elements and SRQR/COREQ (Standards for Reporting Qualitative Research/the Consolidated Criteria for Reporting Qualitative Research) for the qualitative components. Findings will be disseminated through the study website, a webinar, peer-reviewed publications, conference presentations, PPI-led activities and engagement with NHS stakeholders. Results are expected in 2026. Participants will be invited to a webinar and a written lay summary will be available to study participants on request.

## Data sharing plan

Data will be made available on reasonable request and subject to Sponsor approval. Deidentified participant data will be available from the co-chief investigator (0000-0002-1101-2256) and may be used for reanalysis.

## Supplementary material

10.1136/bmjopen-2025-112691 online supplemental file 1

10.1136/bmjopen-2025-112691 online supplemental file 2
