# Supplementary material for: Is home-based self-swabbing feasible for postoperative wound culture after cardiac surgery? A multicentre mixed-methods feasibility study in the UK
Source: BMJ Open. 2026 Feb 10;16(2):e112691. doi: 10.1136/bmjopen-2025-112691 (PMC12911688; doi:10.1136/bmjopen-2025-112691)
Supplement: online supplemental file 1 [file bmjopen-16-2-s001.doc]

**Patient Identification Number for this trial:**

**INFORMED CONSENT FORM - Patient**

**Title:** **A feasibiliTy study to exploRE the safety, Acceptability and potential cost effectiveness of Self-swabbing at home to obtain usable surgical wound cultURE swabs (TREASURE)**

**Name of Researcher:** Please insert researcher name

|  | **Please initial box** |
| --- | --- |
|  | |
| 1. I confirm that I have read and understand the information sheet dated Day/Month/Year (Version x.x) for the above study and have had the opportunity to consider the information, ask questions and have these answered satisfactorily. |  |
|  | |
| 1. I understand that my participation is voluntary and that I am free to withdraw at any time, without giving any reason, without medical care or legal rights being affected. |  |
|  | |
| 1. I understand that relevant sections of any of my medical notes and data collected during the study may be looked at by responsible individuals from the study Sponsor or from regulatory authorities where it is relevant to my taking part in research. I give permission to these individuals to have access to my records. |  |
|  | |
| 1. I agree with the publication of the results of this study in a medical journal (all data will be published anonymously). |  |
|  | |
| 1. I understand that my data will be analysed anonymously, by researchers outside my local hospital and all data will be analysed within the UK |  |
|  | |
| 1. I agree to give wound samples which will be used for analysis by direct culture only. Samples will be sent to external labs in the United Kingdom and after processing will be safely disposed of after three days. I understand I will not be informed of the result. |  |
| 1. I agree to my GP being informed about my participation in this study. |  |
| 1. I agree that my anonymised transcribed interview data will be stored in the University of Nottingham’s (a partner in the study) data repository. |  |
|  | |
| 1. If I choose to stop taking part in the study, the research team can continue collecting information about my surgical wound from central NHS records/ my hospital/ my GP until 30 days after my surgery. (Optional) |  |
| 1. I agree to take part in the above study |  |
|  | |

________________________ ________________ ____________________

Name of Patient Date Signature

_________________________ ________________ ____________________

Name of person taking consent Date Signature

***When completed 1 for patient; 1 for researcher; 1 (original) to be kept with hospital notes***
