# Supplementary material for: Is home-based self-swabbing feasible for postoperative wound culture after cardiac surgery? A multicentre mixed-methods feasibility study in the UK
Source: BMJ Open. 2026 Feb 10;16(2):e112691. doi: 10.1136/bmjopen-2025-112691 (PMC12911688; doi:10.1136/bmjopen-2025-112691)
Supplement: online supplemental file 2 [file bmjopen-16-2-s002.doc]

**Patient Identification Number for this trial:**

**INFORMED CONSENT FORM - Carer**

**Title:** **A feasibiliTy study to exploRE the safety, Acceptability and potential cost effectiveness of Self-swabbing at home to obtain usable surgical wound cultURE swabs (TREASURE)**

**Name of Researcher:** Please insert researcher name

|  | **Please initial box** |
| --- | --- |
|  | |
| 1. I confirm that I have read and understand the information sheet dated Day/Month/Year (Version x.x) for the above study and have had the opportunity to consider the information, ask questions and have these answered satisfactorily. |  |
|  | |
| 1. I understand that my participation is voluntary and that I am free to withdraw at any time, without giving any reason |  |
|  | |
| 1. I understand that relevant sections of any of my data collected during the study may be looked at by responsible individuals from the study Sponsor or from regulatory authorities where it is relevant to my taking part in research. I give permission to these individuals to have access to my research data. |  |
|  | |
| 1. I agree with the publication of the results of this study in a medical journal (all data will be published anonymously). |  |
|  | |
| 1. I understand that my data will be analysed anonymously, by researchers outside my local hospital and all data will be analysed within the UK |  |
|  | |
| 1. I agree that my anonymised transcribed interview data will be stored in the University of Nottingham’s (a partner in the study) data repository. |  |
|  | |
| 1. I agree to take part in the above study |  |
|  | |

The name of the patient participant I am caring for in this study is __________________________

________________________ ________________ ____________________

Name of Carer Date Signature

_________________________ ________________ ____________________

Name of person taking consent Date Signature

***When completed 1 for carer; 1 for researcher; 1 (original) to be kept with hospital notes***
