# Supplementary material for: Is home-based self-swabbing feasible for postoperative wound culture after cardiac surgery? A multicentre mixed-methods feasibility study in the UK
Source: BMJ Open. 2026 Feb 10;16(2):e112691. doi: 10.1136/bmjopen-2025-112691 (PMC12911688; doi:10.1136/bmjopen-2025-112691)
Supplement: Supplementary file 3 [file bmjopen-2025-112691.reviewer_comments.pdf]

PEER REVIEW HISTORY

BMJ Open publishes all reviews undertaken for accepted manuscripts. Reviewers are asked to complete a checklist review form and are provided with free text boxes to elaborate on their assessment. These free text comments are reproduced below.

ARTICLE DETAILS

Title (Provisional)

Protocol for a feasibility study to explore the safety, acceptability and potential costeffectiveness of self-swabbing at home for surgical wound culture (TREASURE)

Authors

Rochon, Melissa; Tanner, Judith; Cariaga, Karen; Harris, Roy; WILSON, KEITH; Newby, Chris; Kariwo, Kumbi; Sowole, Luciana; Bolton, Sarah J; Bouttell, Janet; Ahmed, Ishtiaq

---

VERSION 1 - REVIEW

**Reviewer**	**1**
**Name**	**Wagner, Adam**
**Affiliation**	**NIHR Applied Research Collaboration (ARC) East of England (EoE)**
**Date**	**18-Nov-2025**
**COI**	**None**

Thank you for the opportunity to review this paper in an interesting and potentially fruitful area. The core question – can patients self-swab – and their views around this make for a consistent narrative/plan. Outside of this, not all of the outcomes/analysis was suitably detailed for me. For example, apparently the swabs will be cultured – but what will be done with this information? The health economic analysis receives one line – some further detail is needed. I hope my feedback below helps refine the paper.

Minor, but important: I was not entirely clear from the introduction where the swab would be processed – will this continue to be in primary care? Or this be at hospital etc? In which case, the intervention is potentially not /just/ the self-swabbing, but change in place of processing? Please clarify.

Please provide some further detail on the health economic analysis on P9, L8/9 – how will current pathways be determined? What perspective do you intend to take? NHS, or more specific?

P7, L46-47: “culture and antimicrobial susceptibility testing” is mentioned in the intervention section – however, nothing seems to be being done with this data – is it being analysed? It does not appear mentioned in the outcomes section.

P8, L41: For me, there is a lack of detail on “laboratory suitability” – how will this be judged – please detail. Is this an objective measure?

P8 - L24-33, L55 in some places a single primary outcome is noted, while in others, multiple primary outcomes are noted – please standardise.

I note from the trial registration webpage: “These will be managed by providing clear step-by-step instructions, video support with a registered nurse, and following the study’s distress protocol.” – mentioned is not made of the registered nurse in the protocol – is this the research on teams? This should be clarified.

Please check carefully that the SPIRIT checklist is filled in appropriately, perhaps utilising “N/A” in more places. For example, 27c (missing data) I don’t think is addressed in your manuscript (a search for “missing” does not result in finding anything).

Minor:

P2, L23-24: Apologies if a silly question, but I am unclear when/how the “if indicated” applies in “Swabs will be couriered to a central laboratory for bacterial culture and antimicrobial susceptibility, if indicated” – does the researcher on teams determine if the wound looks infected, and only if positive, is the swab couriered? (Apologies, this may make sense if one is more familiar with the area)

P2, L29: I am unconvinced “health economic analysis” can be considered an outcome – can you reframe?

P5, L18-32: So irrespective of suspected infection status, all patients will try to swab their wound?

P5, L35-39: Perhaps here, or elsewhere, motivate/justify briefly the cardiac setting? (I have no problem with this focus – a justification would be useful if there is one). Are SSIs more common in this setting?

P5, L45-48: Have you anything in place for non-adult carers?

P5: Participant criteria – criteria not given for carer?

P6, L12: I appreciate the challenge of a dressing to your research question, but it seems odd to apply a criteria that may happen after initial recruitment? Indeed, is this a useful parameter for your future trial – a subset of patients for whom swabbing can’t be used?

P6, L14-20: For non-clinicians, perhaps add a few words to motivate these latter exclusions?

P6, L24-26: For precision specification in feasibility studies, I would expect to assume 0.5/50% to get the widest 95% CI. A brief comment to justify the high success rate would be useful.

P6, L27/28: In line with this being a feasibility study and the sentence on L24 (“As a feasibility study, no formal hypothesis testing is planned.”), I find it odd to read of statistical power – can this sentence be removed, or replaced with a precision estimate?

P7, L25/26: Staff eligibility criteria are not covered. Please add.

P7, L31-52: A researcher will be observing a chest wound on teams – perhaps a brief comment to note that the researcher took these Teams call in a private setting for the comfort/privacy of patients?

P9, L42/43: I am not a clinician, but it is not immediately obvious to me that the study is low risk – I fully concede I wouldn’t consider it high risk, but you are proposing something (a swab) gets introduced to a surgical wound – perhaps a brief comment to reassure on this rating?

Very minor:

P1, L51: Double space between colon and “Mr”

P4, L39: Double space following full stop.

P5, L18-32: Perhaps a sentence at the end of the main paragraph to motivate/introduce the health economic work?

P5, L31/32: Space needed between reference and “The”: “[21]The”

P6, L56: First use of ICF and PIF – please spell out.

P8, L5: Perhaps “stored using REDCap” would be more apt – make it clear that there are no REDCap forms being completed by the patient?

P10, L37-44: Various acronyms introduced for the first time should be spelt out.

P12, L10: Missing detail? “management. . London” (e.g. two full stops).

VERSION 1 - AUTHOR RESPONSE

Reviewer: 1
Dr. Adam Wagner, NIHR Applied Research Collaboration (ARC) East of England (EoE), University of East Anglia
Comments to the Author:
Thank you for the opportunity to review this paper in an interesting and potentially fruitful area. The core question – can patients self-swab – and their views around this make for a consistent narrative/plan. Outside of this, not all of the outcomes/analysis was suitably detailed for me. For example, apparently the swabs will be cultured – but what will be done with this information? We have clarified that susceptibility testing will not form part of the secondary outcomes, however antimicrobial susceptibility result data will be collected as part of normal testing processes. The health economic analysis receives one line – some further detail is needed. We agree, this was too

limited and we have provided additional information on HE. I hope my feedback below helps refine the paper. Thank you, we feel the work is strengthened as a result of your review.

Minor, but important: I was not entirely clear from the introduction where the swab would be processed – will this continue to be in primary care? Or this be at hospital etc? In which case, the intervention is potentially not /just/ the self-swabbing, but change in place of processing? Please clarify. We have clarified to say that swabs will be processed in an independent lab used by the NHS. This is not an entirely different process as some patients may have come back to hospital clinics to have wounds swabbed.

Please provide some further detail on the health economic analysis on P9, L8/9 – how will current pathways be determined? What perspective do you intend to take? NHS, or more specific? We have clarified that this will be from an NHS health and personal social services perspective.

P7, L46-47: "culture and antimicrobial susceptibility testing" is mentioned in the intervention section – however, nothing seems to be being done with this data – is it being analysed? It does not appear mentioned in the outcomes section. Thank you for highlighting this point. Culture and antimicrobial susceptibility testing will be performed (if a pathogen is cultured), but does not form part of the analysis under a secondary outcome.

P8, L41: For me, there is a lack of detail on "laboratory suitability" – how will this be judged – please detail. Is this an objective measure? Laboratory suitability is about whether the sample is appropriate to be tested. We have changed the wording to reflect this

P8 - L24-33, L55 in some places a single primary outcome is noted, while in others, multiple primary outcomes are noted – please standardise. We have standardised the primary outcomes across trial registry information, protocol and manuscript.

I note from the trial registration webpage: "These will be managed by providing clear step-by-step instructions, video support with a registered nurse, and following the study's distress protocol." – mentioned is not made of the registered nurse in the protocol – is this the research on teams? This should be clarified. This is referring to the research practitioner in MS Teams. Thank you. We have requested to update our study records in both trial registries. These are currently in progress.

Please check carefully that the SPIRIT checklist is filled in appropriately, perhaps utilising "N/A" in more places. For example, 27c (missing data) I don't think is addressed in your manuscript (a search for "missing" does not result in finding anything). SPIRIT checklist updated

Minor:

P2, L23-24: Apologies if a silly question, but I am unclear when/how the "if indicated"

applies in “Swabs will be couriered to a central laboratory for bacterial culture and antimicrobial susceptibility, if indicated” – does the researcher on teams determine if the wound looks infected, and only if positive, is the swab couriered? (Apologies, this may make sense if one is more familiar with the area) Thank you for the opportunity to clarify – antibiotic susceptibility is only indicated if potential pathogen has been cultured (grown). We hope the addition from P7, L46-47: helps to clarify.

P2, L29: I am unconvinced “health economic analysis” can be considered an outcome – can you reframe? Redone.

P5, L18-32: So irrespective of suspected infection status, all patients will try to swab their wound? The inclusion/exclusion criteria applies- i.e. the wound must be closed without appearance of infection.

P5, L35-39: Perhaps here, or elsewhere, motivate/justify briefly the cardiac setting? (I have no problem with this focus – a justification would be useful if there is one). Are SSIs more common in this setting? Rationale provided and referenced.

P5, L45-48: Have you anything in place for non-adult carers? We will not be recruiting participants who have non-adult carers.

P5: Participant criteria – criteria not given for carer? As above.

P6, L12: I appreciate the challenge of a dressing to your research question, but it seems odd to apply a criteria that may happen after initial recruitment? Indeed, is this a useful parameter for your future trial – a subset of patients for whom swabbing can’t be used? We will be collecting this data and will report this in future using CONSORT.

P6, L14-20: For non-clinicians, perhaps add a few words to motivate these latter exclusions? Thank you, we have added due to potential issues with healing.

P6, L24-26: For precision specification in feasibility studies, I would expect to assume 0.5/50% to get the widest 95% CI. A brief comment to justify the high success rate would be useful. Added-90% was drawn as part of a Green/Go criteria for a full trial.

P6, L27/28: In line with this being a feasibility study and the sentence on L24 (“As a feasibility study, no formal hypothesis testing is planned.”), I find it odd to read of statistical power – can this sentence be removed, or replaced with a precision estimate? The sentence has been removed.

P7, L25/26: Staff eligibility criteria are not covered. Please add. Added.

P7, L31-52: A researcher will be observing a chest wound on teams – perhaps a brief comment to note that the researcher took these Teams call in a private setting for the comfort/privacy of patients? Statement added.

P9, L42/43: I am not a clinician, but it is not immediately obvious to me that the study is low risk – I fully concede I wouldn’t consider it high risk, but you are proposing something (a swab) gets introduced to a surgical wound – perhaps a brief comment to reassure on this rating? Added, including supervision of all steps including hand hygiene by the researcher.

Very minor:
P1, L51: Double space between colon and “Mr” Amended
P4, L39: Double space following full stop. Amended
P5, L18-32: Perhaps a sentence at the end of the main paragraph to motivate/introduce the health economic work? Amended
P5, L31/32: Space needed between reference and “The”: “[21]The” Amended
P6, L56: First use of ICF and PIF – please spell out. Amended
P8, L5: Perhaps “stored using REDCap” would be more apt – make it clear that there are no REDCap forms being completed by the patient? Amended
P10, L37-44: Various acronyms introduced for the first time should be spelt out. Amended
P12, L10: Missing detail? “management. . London” (e.g. two full stops). Amended

Reviewer: 1
If you have selected ‘Yes’ above, please provide details of any competing interests.: Not applicable
